# LncRNA LOXL1-AS1 Promotes the Proliferation and Metastasis of Medulloblastoma by Activating the PI3K/AKT Pathway

**DOI:** 10.1155/2018/9275685

**Published:** 2018-06-27

**Authors:** Ran Gao, Rui Zhang, Cuicui Zhang, Yingwu Liang, Weining Tang

**Affiliations:** ^1^Department of Pediatrics, Jining No. 1 People's Hospital, Shandong, China; ^2^Department of Pediatrics, Sishui People's Hospital, Shandong, China; ^3^Intensive Care Unit, Affiliated Hospital of Jining Medical University, Shandong, China

## Abstract

Medulloblastoma is the most common malignant brain tumor of childhood, with great potential to metastasize. However, the mechanisms of how medulloblastoma develops and progresses remain to be elucidated. The present study assessed the role of long noncoding RNA LOXL1-AS1 (lncRNA LOXL1-AS1) in the cell proliferation and metastasis in human medulloblastoma. It was initially found that LOXL1-AS1 was significantly overexpressed in clinical medulloblastoma tissues compared with the adjacent noncancerous tissues. LOXL1-AS1 was also highly expressed in medulloblastoma at advanced stages and differentially expressed in a series of medulloblastoma cell lines. Knockdown of LOXL1-AS1 using shRNAs significantly inhibited cell viability and colony formation capacities in D283 and D341 cells. Moreover, the cell proportion in the S phase was significantly increased, while the cell proportion in the G2/M phase was decreased after knockdown of LOXL1-AS1 in D283 cells and D341 cells. Cell cycle arrest led to eventual cell apoptosis by LOXL1-AS1 knockdown. Moreover, in a xenograft model of human medulloblastoma, knockdown of LOXL1-AS1 significantly inhibited tumor growth and promoted tumor cell apoptosis. In addition, knockdown of LOXL1-AS1 inhibited cell migration and reversed epithelial-to-mesenchymal transition (EMT). Western blot analysis further revealed that knockdown of LOXL1-AS1 decreased the phosphorylated levels of PI3K and AKT without affecting their total protein levels. These results suggest that LncRNA LOXL1-AS1 promoted the proliferation and metastasis of medulloblastoma by activating the PI3K-AKT pathway, providing evidence that knockdown of LncRNA LOXL1-AS1 might be a potential therapeutic strategy against medulloblastoma.

## 1. Introduction

Medulloblastoma is the most common malignant brain tumor of childhood characterized with frequent extraneural metastasis [1]. Current therapies for medulloblastoma were introduced primarily in the 1980s and consist of predominantly cytotoxic, nontargeted approaches. However, mortality from medulloblastoma remains significant [2]. Moreover, many survivors suffer from severe treatment-related effects of radiation and cytotoxic chemotherapy such as endocrinological dysfunction and intellectual damage [3, 4]. Therefore, novel therapeutic strategies targeting critical regulatory pathways in the development and progression of medulloblastoma are warranted.

Currently, the origin of cancer is considered as a step-by-step accumulation of alterations in cell function and molecular expression, which are widely reported to relate with mechanisms involving transcriptional regulation [5], posttranscriptional regulation [6], and epigenetic modification [7]. Among the posttranscriptional regulatory machineries, long noncoding RNAs (lncRNAs) have recently been identified as key regulators of various biological processes, including cell proliferation, differentiation, apoptosis, migration, and invasion [8–10]. lncRNAs are a class of RNA over 200 nucleotides in length. The role of lncRNAs in solid tumors has received increasing attention from worldwide studies. Moreover, lncRNAs, such as SNHG1, have been associated with cancer malignancy in pan-cancer including medulloblastoma [11]. However, our knowledge of lncRNAs remains limited, and it has become a major research challenge in discovering novel disease-related lncRNAs in cancers such as medulloblastoma [11].

Emerging data has shown the critical role of lncRNAs in the development and progression of medulloblastoma. Tumor growth and metastasis of medulloblastoma have been reported to be strictly controlled by lncRNAs such as CCAT1 [10], linc-NeD125 [12], and CRNDE [9]. However, other critical lncRNAs significantly associated with medulloblastoma remain to be elucidated.

lncRNA LOXL1-antisense RNA (LOXL1-AS1) is encoded on the opposite strand of LOXL1. It is a novel lncRNA that has recently been identified using sequencing and genetic analysis [13]. LOXL1-AS1 expression is significantly altered in response to oxidative stress in human lens epithelial cells and in response to cyclic mechanical stress in human Schlemm's canal endothelial cells [13], supporting a functional role for the lncRNA LOXL1-AS1 in cellular stress response.

The role of LOXL1-AS1 in human tumorigenesis remains unknown, so the present study aimed to investigate the expression profile and functional role of LOXL1-AS1 in medulloblastoma. To this end, the LOXL1-AS1 level was initially evaluated in clinical medulloblastoma tissues and in a series of medulloblastoma cell lines. Specific shRNAs targeting LOXL1-AS1 were then synthesized to modulate the expression of LOXL1-AS1. Cell viability, colony formation, and cell migration capacities were examined *in vivo* and *in vitro*. Our results showed that LOXL1-AS1 was highly expressed in medulloblastoma tissues. Knockdown of LOXL1-AS1 significantly inhibited cell proliferation and metastasis in medulloblastoma. In addition, the PI3K/AKT pathway was regulated by LOXL1-AS1, which might suggest a regulatory mechanism contributing to LOXL1-AS1-mediated medulloblastoma progression.

## 2. Materials and Methods

### 2.1. Human Tissues and Ethical Statements

A total of 50 cases that were clinically diagnosed with medulloblastoma at Jining No. 1 People's Hospital and Sishui People's Hospital were included in the present study. For each case, its cancerous tissues and the matched adjacent noncancerous tissues were obtained. All patients showed their full consent to participate in our study, and a written consent form was obtained from each patient. Protocols for using human tissues were approved by the ethical committee board at Jining No. 1 People's Hospital and Sishui People's Hospital University.

### 2.2. Cells and Reagents

Human medulloblastoma cell lines Daoy, D283, D425, D341, and D458 were purchased from the Cell Bank of the Chinese Academy of Sciences (Shanghai, China). All cell lines were maintained in Dulbecco's Modified Eagle Medium (DMEM) (Gibco, Los Angeles, CA, USA) supplied with 10% fetal bovine serum (FBS) (Gibco). Culture medium was refreshed every two days unless otherwise stated. Primary antibodies were commercially purchased from Santa Cruz Biotechnology (Santa Cruz, CA, USA) except for the phosphorylation detection antibodies which were obtained from Cell Signaling Technology (Boston, MA, USA). For knockdown of lncRNA LOXL1-AS1, two specific shRNAs were chemically synthesized by GenePharma (Shanghai, China). A scramble shRNA was also synthesized serving as control shRNA.

#### 2.2.1. Quantitative Real-Time PCR

Total RNAs of human tissues and cultured cells were isolated using TRIzol Reagent (Thermo Fisher Scientific, Waltham, MA, USA) according to the manufacturer's instruction. The quality and concentration of extracted RNAs were determined by collecting the absorbance at 260 nm with Nanodrop 2000. The RNAs were immediately transcribed into cDNAs using the PrimeScript RT Master Mix Perfect Real Time (TaKaRa, Shiga, Japan). All real-time PCRs were performed with the SYBR Premix Ex Taq Kit (TaKaRa, Japan) in an ABI PRISM 7500 Real-Time System. *Glyceraldehyde-3-phosphate dehydrogenase (GAPDH)* was included as the internal control. Each experiment was repeated three times with each one performed in triplicate.

### 2.3. Western Blot Analysis

Total proteins were extracted using a RIPA lysis buffer (pH = 7.5, Beyotime Biotechnology, Nantong, China) to generate the whole protein lysate. An equal amount of 40 *μ*g protein was loaded to each lane in a 12% SDS-PAGE gel. Proteins were then transferred to a polyvinylidene fluoride (PVDF) membrane after electrophoresis. After blockade of nonspecific antigens with 5% skim milk, membranes were incubated with primary antibodies overnight at 4°C. A secondary antibody which recognizes the primary antibody was then added, and the immunoreactivity was determined with enhanced chemoluminescent autoradiography (Thermo Scientific, PA, USA). GAPDH was synchronously developed for loading control.

### 2.4. Immunofluorescent Assay

D283 and D341 cells with indicated treatments were seeded on sterile coverslips in a 24-well plate in DMEM with 10% FBS. After 24 h, cells were rinsed with PBS and fixed for 30 min in 4% paraformaldehyde. Subsequently, cells were penetrated with 2% Triton X-100 for 20 min and then blocked in 5% FBS for 1 h. After that, cells were incubated with primary antibodies at 4°C overnight. After washing by PBS, cells were incubated with corresponding secondary antibody in the dark for 1 h and counterstained for 30 min with 4′, 6-diamidino-2-phenylindole (DAPI). Samples were finally mounted and observed/photographed by an Olympus IX71 inverted microscope (Olympus Optical Co., Tokyo, Japan).

### 2.5. Cell Viability Detection

D283 and D341 cell viability were assessed using a cell counting kit-8 (CCK-8) assay (Beyotime, Nantong, China) according to the manufacturer's protocol. Briefly, cells were seeded into 6 cm dishes and transfected with specific shRNAs against LOXL1-AS1 (shRNA1 or shRNA2 group) or with a scramble shRNA (control group). Twenty-four hours later, each group of cells were trypsinized and resuspended. Cells were then seeded into a 96-well plate at an initial concentration of 8000 cells per well. Cell proliferative rates were monitored for the following 5 days. On each monitored time point, an aliquot of 10 *μ*l of CCK-8 solution was added to each well. After further incubation of cells with CCK-8 solution for 4 h, the absorbance of each well was measured by an ELISA reader at a wave length of 450 nm. For data analysis, the cell viability on day 1 was set as 1.

### 2.6. Colony Formation Assay

Human medulloblastoma cell lines D283 and D341 were pretransfected with specific shRNA against LOXL1-AS1 and spread into 12-well plates. Then, all plates were incubated for 2 weeks to allow colony formation. After that, colonies were stained with crystal violet (0.1%) for 30 min. A colony is defined as cell accumulation with over 50 cells. The total number of colonies was manually counted and averaged for each group.

### 2.7. Wound-Healing Assay and Transwell Migration Assay

For the wound-healing assay, cells were plated on 6-well plates to form a confluent monolayer. Wounds were made with sterile pipette tips. Wound recovery was observed every 6 hours. The wound recovery rate was then calculated after each monitored time point. A migration assay was carried out using Boyden chambers (tissue culture-treated, 6.5 mm diameter, 8 *μ*m pores, Transwell, Costar, Cambridge, MA, USA) containing polycarbonate membrane. Briefly, 100 *μ*l of 1 × 10^6^ cells in serum-free medium was added to the upper chamber, and 600 *μ*l of DMEM with 10% FBS was added to the lower chamber. Cells were incubated for 12 h. Migrated cells on the undersurface of the membrane were fixed and stained with crystal violet for 10 minutes at room temperature. Photographs of five random regions were taken, and the number of cells was counted to calculate the average number of migrated cells per plate.

### 2.8. Cell Cycle Analysis

Flow cytometry analysis was performed to analyze cell cycle progression. Briefly, control or LOXL1-AS1-depleted D283 cells and D341 cells were washed with PBS twice and fixed with 70% ethanol for 30 min on ice. To degrade RNAs, 20 mg/ml of RNase (Sigma-Aldrich, NY, USA) was added for 1 h at 37°C. After RNA degradation, samples were then stained with 20 mg/ml propidium iodide (PI, Sigma-Aldrich), and cell proportion at each phase was assessed by FACSCalibur flow cytometry (BD Biosciences, San Jose, CA, USA) equipped with the ModiFit LT v2.0 software.

### 2.9. Cell Apoptosis Analysis

Cell apoptosis was analyzed using the Annexin V/PI apoptosis kit (Invitrogen, Shanghai, China) according to the manufacturer's instruction. Briefly, D283 and D341 cells were seeded in a 6-well plate (2 × 10^6^ cells/well) and transfected with a scramble shRNA (control group) or with the specific shRNA against LOXL1-AS1 (shRNA group). Both cell lines were then cultured with complete medium for 48 h. After that, cells were washed with iced PBS and resuspended with 100 *μ*l binding buffer. Then, 5 *μ*l of annexin V-FITC and 5 *μ*l of PI working solution (100 μg/ml) were added to the 100 *μ*l aliquots of cell suspension. Cell suspensions were further incubated at room temperature for 15 min in the dark. Subsequently, another 400 *μ*l of binding buffer was added to the cell suspension. Samples were then analyzed by flow cytometry. Each sample was tested in triplicate three times.

### 2.10. Xenograft Model of Medulloblastoma *In Vivo*

Male athymic BALB/c nude mice that were 5 weeks old were maintained in a special pathogen-free (SPF) condition. A total of 10 mice were fed and randomly divided into the control or shRNA group (*n* = 5 per group). D283 cells were pretransfected with the scramble shRNA (control) or specific shRNA1 against LOXL1-AS1 (shRNA1 group) prior to inoculation into mice. A total of 5 × 10^6^ D283 cells with indicated treatments were then injected subcutaneously into the right flank in each mouse. Tumor dimensions (length, *L*, and width, *W*) were then measured once a week for a total of 4 weeks. Tumor volumes (TV) were calculated as TV = *L* × *W*^2^/2. After 4 weeks feeding, the mice were sacrificed and tumor tissues were dissected for subsequent analyses. All efforts were made to minimize suffering.

### 2.11. Histology and Immunohistochemistry (IHC) Analysis

Tumor tissues from the mouse model were paraffin embedded and cut into 4 *μ*m slides. The slides were then subject to hematoxylin and eosin (H&E) staining for pathology confirmation. After H&E staining, the slides were subjected to antigen retrieval in a microwave in 0.1 M citric acid solution (pH 6.0) for 10 min. After incubation with primary antibodies at 4°C overnight, the slides were incubated with corresponding secondary antibody at room temperature for 1 h. The reactivity was developed using 0.05% diaminobenzidine (DAB) containing 0.01% H_2_O_2_. Representative images were photographed for each slide.

### 2.12. Statistical Analysis

Data were expressed as means ± standard deviation (SD). Comparisons between groups were analyzed using Student's *t*-test. Differences with a two-sided *p* value < 0.05 were considered to be statistically significant.

## 3. Results

### 3.1. lncRNA LOXL1-AS1 Was Overexpressed in Medulloblastoma

**Figure 1:**
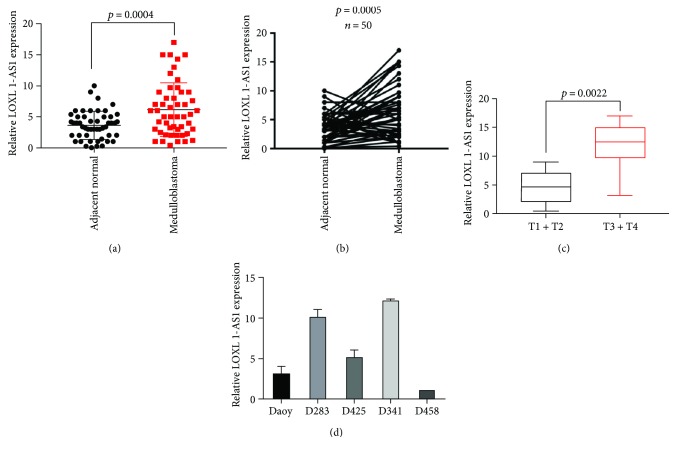
lncRNA LOXL1-AS1 was highly expressed in medulloblastoma. (a) qRT-PCR analysis of LncRNA LOXL1-AS1 in 50 cases of clinical medulloblastoma as well as their adjacent normal tissues. (b) The paired expression of LOXL1-AS1 was shown in the 50 cases. (c) All the 50 cases were subgrouped as T1, T2, T3, and T4 based on the tumor size. Relative level of LOXL1-AS1 in T3 and T4 cancerous tissues was significantly higher than that in T1 and T2 tissues. (d) qRT-PCR analysis of the relative level of LOXL1-AS1 in 5 medulloblastoma cell lines. The level of LOXL1-AS1 in the D458 cells was set as 1. LOXL1-AS1 levels in other cell lines were normalized to those in D458 cells. The *p* value for each comparison was indicated in each panel.

Initially, the expression of LOXL1-AS1 was examined in clinical medulloblastoma tissues. In the 50 cases, the mean level of LOXL1-AS1 in medulloblastoma tissues was approximately 1.5-fold that in the adjacent noncancerous tissues (Figure 1(a)). After analysis of the paired tissues, it was found that 36 of the 50 cases showed a higher level of LOXL1-AS1 in medulloblastoma tissues as compared with the paired adjacent tissues (Figure 1(b)). Moreover, the relative LOXL1-AS1 level was significantly higher in tumors with the size categorized as T3 and T4 (Figure 1(c)). In a series of medulloblastoma cell lines, LOXL1-AS1 was differentially expressed and showed the highest levels in D283 and D341 cells (Figure 1(d)). Taken together, these data suggest that LOXL1-AS1 was overexpressed in medulloblastoma tissues.

### 3.2. Knockdown of LOXL1-AS1 Inhibited Cell Viability and Colony Formation Capacity in D283 and D341 Cells

**Figure 2:**
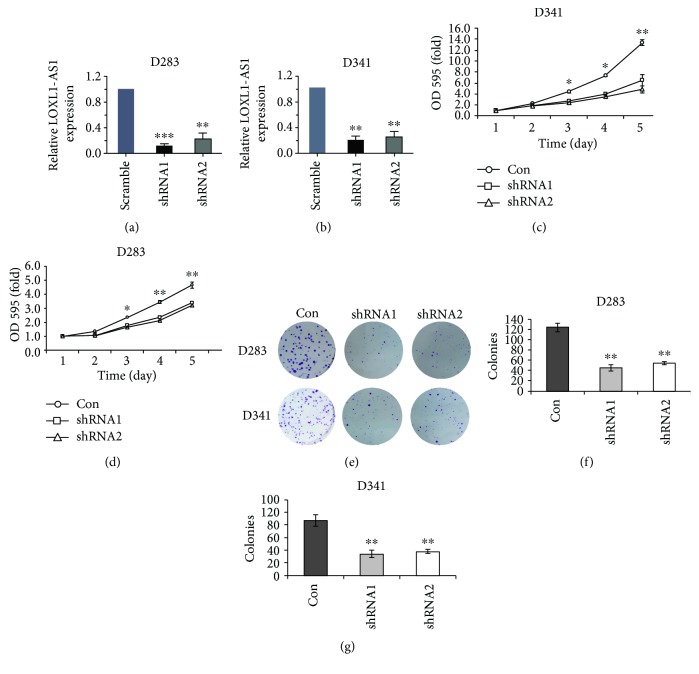
Knockdown of LOXL1-AS1 inhibited cell viability and colony formation capacity in D283 and D341 cells. (a, b) Knockdown efficiency of two synthesized shRNAs against LOXL1-AS1 (termed as shRNA1 and shRNA2) was assessed in D283 cells and D341 cells, respectively. (c, d) After knockdown of LOXL1-AS1 in D283 and D341 cells, cell proliferative rates were monitored in a consecutive of 5 days. The absorbance on day 1 was set as 1 for each group of cells. (e, f, g) Control and LOXL1-AS1-depleted cells were subject to colony formation assay in D283 cells and D341 cells. Formed colonies were stained with crystal violet, and all colonies in each group were manually counted and averaged from three independent assays. ^∗^*p* < 0.05; ^∗∗^*p* < 0.01.

In view of the highest expression of LOXL1-AS1 in D283 and D341 cells, these two cell lines were selected as optimal to investigate the functional roles of LOXL1-AS1 in medulloblastoma. Specific shRNAs against LOXL1-AS1 were synthesized. It was shown that specific shRNAs depleted the expression of LOXL1-AS1 in D283 cells (Figure 2(a)) and D341 cells (Figure 2(b)). Next, cell viability was determined in both cell lines with or without LOXL1-AS1 depletion. In D283 cells, it was observed that depletion of LOXL1-AS1 impaired cell proliferative abilities from day 3. On day 5, the proliferative rate in LOXL1-AS1-depleted cells was only half of that in control cells (Figure 2(c)). Depletion of LOXL1-AS1 using either shRNA also inhibited cell proliferation by up to 70% in D341 cells (Figure 2(d)). Likewise, in the colony formation assay, it was visually observed that depletion of LOXL1-AS1 in either cell line decreased the colonies that were stained with crystal violet (Figure 2(e)). Quantification of the colonies showed that only approximately 40–50 colonies were formed in LOXL1-AS1-depleted D283 cells, which was in contrast to the mean 120 colonies in control D283 cells (Figure 2(f)). Decreases in the formed colonies were also found in LOXL1-AS1-depleted D341 cells (Figure 2(g)). All these data suggested that knockdown of LOXL1-AS1 inhibited cell viability and clonogenic potential in medulloblastoma cells.

### 3.3. Knockdown of LOXL1-AS1 Arrested Cell Cycle at the S Phase and Led to Eventual Cell Apoptosis in Medulloblastoma

**Figure 3:**
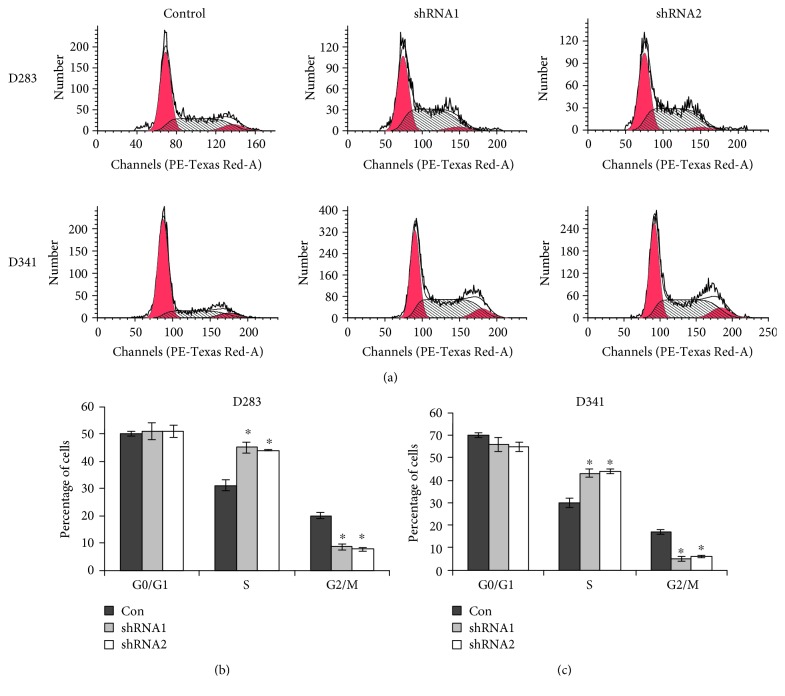
Knockdown of LOXL1-AS1 arrested cell cycle at the S phase in medulloblastoma. (a) Cell cycle progression was analyzed in both D283 cells and D341 cells that were pretreated with or without specific shRNAs against LOXL1-AS1. (b, c) Cell proportions in the G0/G1, S, and G2/M phases were calculated in D283 cells and D341 cells, respectively. It was found that cells were proportionally accumulated in the S phase while cells in the G2/M phase were significantly decreased in both cell lines. ^∗^*p* < 0.05.

The effects of LOXL1-AS1 knockdown were then assessed on cell survival (Figure 3(a)). In the cell cycle analysis, it was found that knockdown of LOXL1-AS1 arrested the cell cycle progression (Figure 3(a)). Particularly, the cell proportion in the S phase was significantly increased from approximately 30% in control D283 cells to nearly 45% in LOXL1-AS1-depleted D283 cells. Accordingly, the cell proportion in the G2/M phase was decreased by approximately 50% after knockdown of LOXL1-AS1 in D283 cells (Figure 3(b)). Comparable cell cycle arrest at the S phase was also observed in D341 cells (Figure 3(c)).

**Figure 4:**
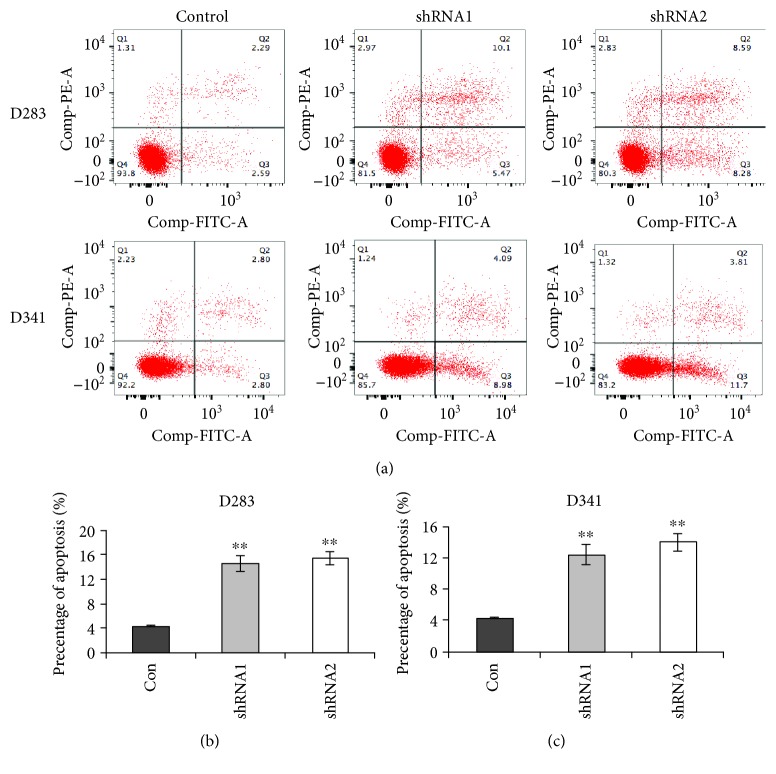
Knockdown of LOXL1-AS1 promoted cell apoptosis in D283 and D341 cells. (a) Cell survival was determined in both cell lines with or without LOXL1-AS1 depletion. (b, c) The percentage of cell apoptosis was shown for D283 cells and D341 cells. It was found that the cell apoptosis was significantly promoted by either shRNA in both cell lines. ^∗∗^*p* < 0.01.

Subsequently, cell apoptosis was analyzed (Figure 4(a)). While there was only approximately 4% control D283 cells that were apoptotic, the cell apoptosis rate was up to 14% in shRNA1-transfected D283 cells and 15.2% in shRNA2-transfected D283 cells (Figure 4(b)). Knockdown of LOXL1-AS1 in D341 also promoted cell apoptosis rates, a 200% increase by shRNA1 and 250% increase by shRNA2 (Figure 4(c)). These data suggest that knockdown of LOXL1-AS1 arrested the cell cycle, leading to eventual cell apoptosis in medulloblastoma.

### 3.4. Depletion of LOXL1-AS1 Inhibited Tumor Growth in Medulloblastoma *In Vivo*

**Figure 5:**
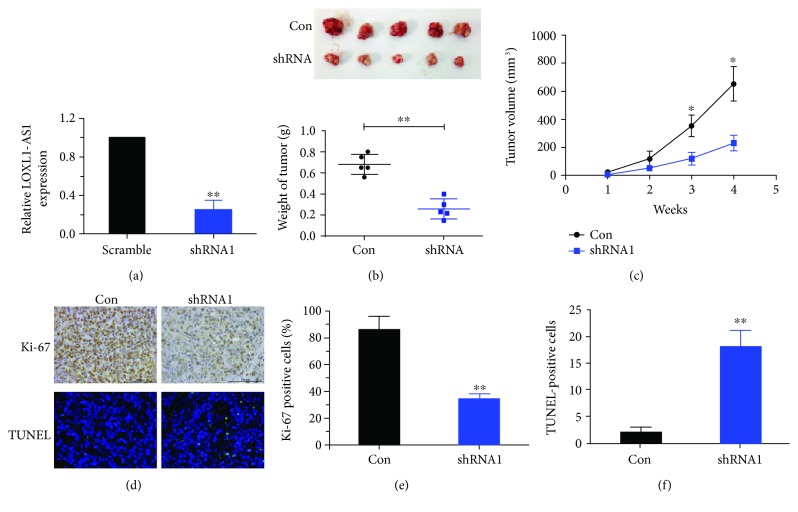
Depletion of LOXL1-AS1 inhibited tumor growth in medulloblastoma *in vivo*. (a) qRT-PCR analysis of relative LOXL1-AS1 levels in D283 cells to confirm the knockdown efficacy of shRNA1 (the first synthesized shRNA against LOXL1-AS1). (b) D283 cells were pretreated with the scramble (control group) or shRNA1 prior to inoculation into mice. Four weeks after inoculation, neoplasia were resected and weighed. (c) During the 4-week monitoring, tumor dimensions were measured and tumor volume was calculated for each group of mice. (d) The neoplasia from control mice or shRNA1-treated mice were subject to immunohistochemistry analysis of Ki-67 (an indicator of cell proliferation) or TUNEL staining (indicating cell apoptosis). (e) The Ki-67-positive cells were calculated for control and shRNA1-treated mice. (f) TUNEL-positive cells were also quantified to show tumor cell apoptosis. ^∗^*p* < 0.05; ^∗∗^*p* < 0.01.

A xenograft model of human medulloblastoma was established by inoculating D283 cells into nude mice. D283 cells were pretreated with a scrambled shRNA or the shRNA1 prior to inoculation. QRT-PCR analysis was performed to confirm the efficiency of the shRNA1 to knockdown LOXL1-AS1 (Figure 5(a)). The use of shRNA1 was due to the observation that shRNA1 exhibited relatively higher efficacy to deplete LOXL1-AS1 than shRNA2 as shown in Figures 2(a) and 2(b). Four weeks after inoculation, tumors in each group of mice were resected, and the shRNA1-treated mice exhibited visibly smaller sized tumors as compared with the control mice. The weight of tumors from the shRNA1 group was also significantly less than that from the control group (Figure 5(b)). Indeed, during the monitored 4 weeks, shRNA1-treated mice began to exhibit smaller tumor sizes from the second week, and LOXL1-AS1-depleted mice showed slower tumor growth rate during the following weeks (Figure 5(c)). Immunohistochemical analysis revealed that the neoplasia from the shRNA1-treated mice showed relatively less positivity of Ki-67, a marker of tumor cell proliferation (Figures 5(d) and 5(e)). Instead, neoplasia from the LOXL1-AS1-depleted mice showed more TUNEL-positive cells (Figures 5(d) and 5(f)), which indicated the increased cell apoptosis within the tissues, supporting the *in vitro* observations and suggesting that LOXL1-AS1 depletion inhibited tumor growth in medulloblastoma.

### 3.5. Knockdown of LOXL1-AS1 Inhibited Cell Migration in Medulloblastoma

**Figure 6:**
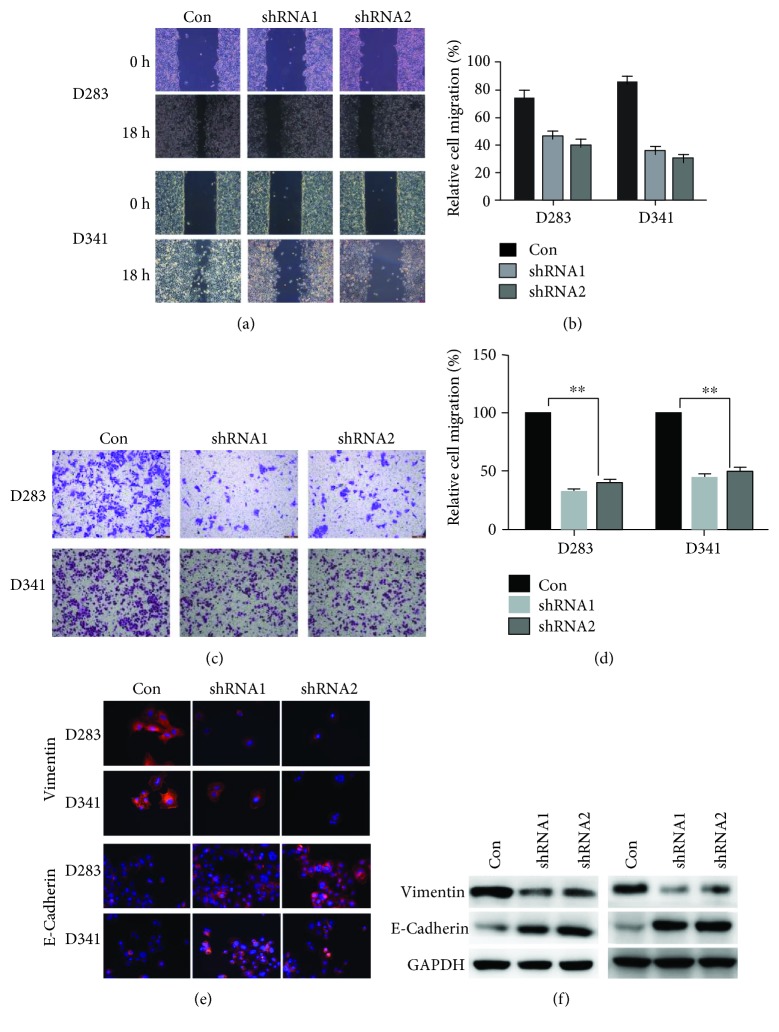
Knockdown of LOXL1-AS1 inhibited cell metastasis and reversed EMT processes in medulloblastoma. (a, b) Control or shRNA-treated D283 and D341 cells were subject to wound-healing assay. Representative images showing the wound recovery were shown at 0 h and 18 h for both cell lines. The wound-recovered area which represented the cell migration capacity was calculated for each group of cells. (c, d) Both D283 and D341 cells were subject to Transwell migration assay. Cells that migrated to the lower surface were stained with crystal violet. Transmigrated cells were counted and averaged from 5 randomly selected fields. (e) Immunofluorescent analysis of vimentin (mesenchymal marker) and E-cadherin (epithelial marker). (f) Western blot analysis of vimentin and E-cadherin in D283 and D341 cells. Expression of vimentin was less detected, whilst that of E-cadherin was largely detected after knockdown of LOXL1-AS1. ^∗^*p* < 0.05; ^∗∗^*p* < 0.01.

Cell migration capacity was then assessed using a wound-healing assay. It was observed that both D283 and 341 cells recovered the artificial wound, regardless of what treatments they received. However, the shRNA-treated cells showed slower recovery ability at 18 h in comparison with control cells (Figure 6(a)). In fact, the wound recovery rates were decreased by nearly 46.7% in D283 cells and 55.6% in D341 cells (Figure 6(b)). The Transwell migration assay also confirmed that LOXL1-AS1-depleted cells were less capable of transmigrating to the lower surface of the chamber (Figure 6(c)). The migrated cells in shRNA-treated groups were less than half of that in control groups (Figure 6(d)).

In addition, the epithelial marker, E-cadherin, and mesenchymal marker, vimentin, were detected, with an increase in E-cadherin immunofluorescence and a decrease in vimentin immunofluorescence observed after knockdown of LOXL1-AS1 (Figure 6(e)). Consistently, Western blot analysis also showed that the protein level of vimentin decreased, whereas that of E-cadherin increased in response to LOXL1-AS1 depletion in both D283 cells and D341 cells (Figure 6(f)), suggesting that knockdown of LOXL1-AS1 inhibited cell migration and reversed EMT processes.

### 3.6. LOXL1-AS1 Positively Regulated the PI3K/AKT Pathway in Medulloblastoma Cell Lines

**Figure 7:**
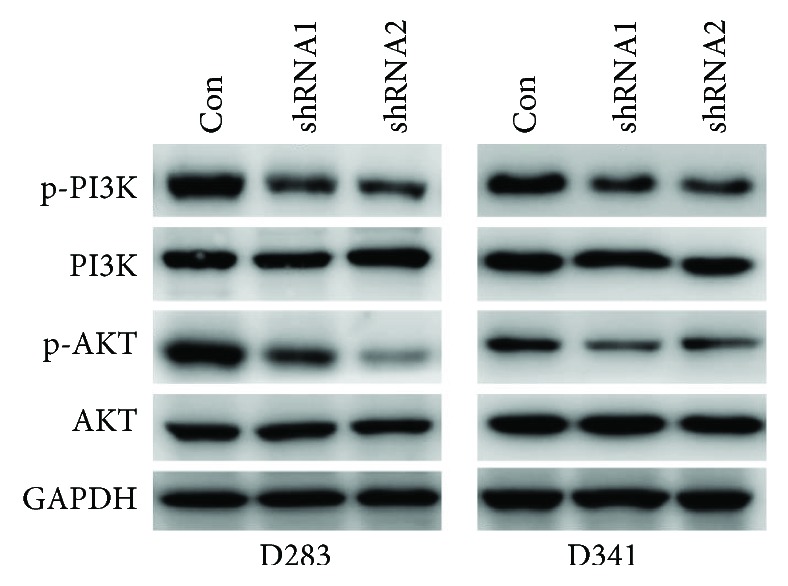
LOXL1-AS1 positively regulated the PI3K/AKT pathway in medulloblastoma cell lines. D283 cells and D341 cells were pretreated with scramble shRNA or specific shRNA1 or shRNA2 before collection of cell lysates. PI3K and AKT were detected at both the dephosphorylated and phosphorylated forms using Western blot analysis.

The molecular mechanisms that contributed to the LOXL1-AS1-mediated phenotype were explored. It was detected that the phosphorylated levels of PI3K (p-PI3K) and AKT (p-AKT) were remarkably decreased in LOXL1-AS1-depleted cells, whereas the total protein levels of PI3K and AKT remained unaffected by LOXL1-AS1 knockdown, indicating that LOXL1-AS1 activated the PI3K/AKT pathway in medulloblastoma (Figure 7).

## 4. Discussion

Medulloblastoma remains a major health problem threating children's lives worldwide. Forty percent of patients suffering from medulloblastoma were found to have distant metastasis at diagnosis [14], making it a real challenge to treat this malignancy. Moreover, traditional therapies for medulloblastoma are associated with significant side effects [3]. Therefore, investigation of novel molecular changes during medulloblastoma initiation and progression is necessary to identify novel therapeutic targets and individualize treatments for medulloblastoma patients.

The present study provided *in vitro* and *in vivo* evidence that LOXL1-AS1 displayed potent prooncogenic functions in medulloblastoma and commends itself as a possible therapeutic target to current medulloblastoma treatment. LOXL1-AS1 is a recently identified lncRNA that is critically associated with cellular stress response [13], but there is a lack of expression data for LOXL1-AS1, especially in tumor tissues. This is the first report that LOXL1-AS1 is significantly overexpressed in clinical medulloblastoma tissues. Its expression was even higher in medulloblastoma with advanced tumor sizes, indicating a critical involvement in tumor growth regulation. In fact, cell viability and clonogenic potential was significantly inhibited after knockdown of LOXL1-AS1 in medulloblastoma cell lines. Tumor growth was also inhibited in an *in vivo* xenografted model of human medulloblastoma with LOXL1-AS1 depletion. Furthermore, the *in vitro* and *in vivo* tumor growth-inhibition effect by LOXL1-AS1 depletion was associated with tumor cell cycle arrest at the S phase. Deregulation of cell cycle progression is a hallmark of tumor growth [15] and closely linked with cell apoptosis [16, 17]. Cell cycle arrest by LOXL1-AS1 depletion supports the tumor growth promotion effect by LOXL1-AS1, as well as the eventual cell apoptosis in D283 and D341 cells, and in the xenografted model of medulloblastoma. Taken together, these observations indicate that LOXL1-AS1 promotes cell growth in medulloblastoma.

In addition, it was also found that knockdown of LOXL1-AS1 impaired cell migration capacities as evidenced by the wound-healing and Transwell migration assays. Cell migration was inhibited by approximately 50% in LOXL1-AS1-depleted D283 cells and D341 cells. EMT is a common manifestation of tumor metastasis and reflects epithelial cell plasticity, in which multiple regulatory molecules are involved, including the Zeb and Snail families [18]. The EMT process is induced by various cellular procedures, including increased expression of mesenchymal markers (N-cadherin and vimentin), decreased protein levels of epithelial markers (E-cadherin), and overexpressed ECM compounds (fibronectin) [19]. EMT is associated with a wide range of human tumorigenesis; activation of EMT in ovarian cancer has been shown to be associated with chemoresistance, which can cause cancer recurrence and metastasis after traditional treatment for ovarian cancer [2, 20, 21]. The present study observed that after depletion of LOXL1-AS1, expression of the mesenchymal marker, vimentin, was downregulated, while the epithelial marker, E-cadherin, was upregulated, suggesting that the EMT process was reversed by LOXL1-AS1 knockdown. Hence, LOXL1-AS1-depleted medulloblastoma cells are more epithelial and less migratory. The EMT assessment supported the wound-healing and Transwell migration assays. Taken together with the tumor growth observations, it can be concluded that LOXL1-AS1 regulates tumor growth and migration in medulloblastoma.

Phosphorylation of PI3K and AKT is pivotal for their activation. Upon phosphorylation, PI3K activates and phosphorylates the downstream AKT to cause a cascade reaction [22]. Interestingly, it was found that the phosphorylated levels of PI3K (p-PI3K) and AKT (p-AKT) were decreased after LOXL1-AS1 depletion, while their total protein levels remained unchanged, suggesting that LOXL1-AS1 positively regulates the PI3K/AKT pathway. PI3K/AKT signaling has been identified as a key driver of cellular proliferation, migration, and angiogenesis in human tumorigenesis, including medulloblastoma, in which activation of PI3K/AKT signaling enhances tumor growth, metastasis, and chemoresistance [23–25]. PI3K/AKT signaling also serves as an integration node in a network of tumor-promoting signal pathways. Inhibition of PI3K activity using the GDC-0941 inhibitor displayed promising *in vitro* and *in vivo* efficacy for targeted medulloblastoma therapy [26]. Targeting the PI3K p110alpha isoform inhibited medulloblastoma proliferation, chemoresistance, and migration [25]. As LOXL1-AS1 promotes cell proliferation and migration in medulloblastoma *via* regulating the PI3K/AKT pathway, any compound or reagent targeting LOXL1-AS1 and consequently inhibiting the PI3K/AKT pathway might serve as a promising therapeutic strategy for the treatment of medulloblastoma.

In summary, the present study identified a novel lncRNA, LOXL1-AS1, as a critical mediator of cell proliferation and migration in medulloblastoma. LOXL1-AS1 is significantly overexpressed in clinical medulloblastoma tissues, while knockdown of LOXL1-AS1 expression impairs tumor cell growth and migration, as well as inactivation of PI3K/AKT. This is the first report of the expression profile and functional role of LOXL1-AS1 in human tumorigenesis, providing strong evidence that a synthetic compound or reagent targeting LOXL1-AS1 or the PI3K/AKT pathway might serve as promising clinical therapeutics against medulloblastoma.
